# On-the-Fly Monitoring of the Capture and Removal of Nanoplastics with Nanorobots

**DOI:** 10.1021/acsnanoscienceau.4c00002

**Published:** 2024-04-09

**Authors:** Dean I. Velikov, Anna Jancik-Prochazkova, Martin Pumera

**Affiliations:** † Future Energy and Innovation Laboratory, Central European Institute of Technology, Brno University of Technology, Purkyňova 123, 612 00 Brno, Czech Republic; ‡ Advanced Nanorobots and Multiscale Robotics Laboratory, Faculty of Electrical Engineering and Computer Science, VSB - Technical University of Ostrava, 17. listopadu 2172/15, 708 00 Ostrava, Czech Republic; § Department of Medical Research, China Medical University Hospital, China Medical University, No. 91 Hsueh-Shih Road, 406040 Taichung, Taiwan

**Keywords:** polystyrene nanoplastics, Nile Red, photoluminescence, magnetic nanorobots, magnetite, removal

## Abstract

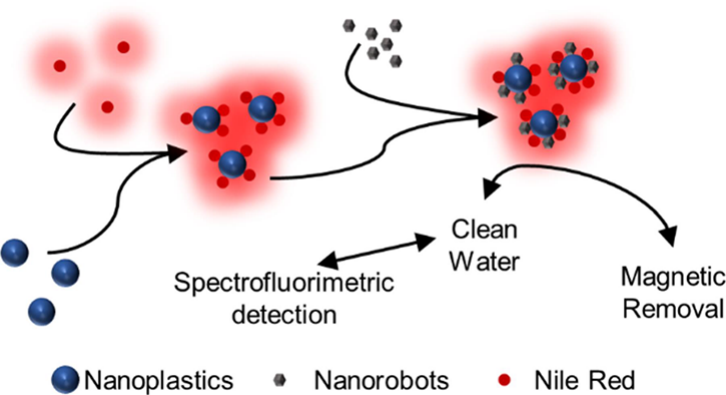

Nanoplastics are considered an emerging organic persistent pollutant with possible severe long-term implications for the environment and human health; therefore, their remediation is of paramount importance. However, detecting and determining the concentration of nanoparticles in water is challenging and time-consuming due to their small size. In this work, we present a universal yet simple method for the detection and quantification of nanoplastics to monitor their removal from water using magnetic nanorobots. Nanoplastics were stained with a hydrophobic fluorescent dye to enable the use of photoluminescence techniques for their detection and quantification. Magnetic nanorobotic tools were employed to capture and subsequently remove the nanoplastics from contaminated waters. We demonstrated that nanorobots can capture and remove more than 90% of the nanoplastics from an aqueous solution within 120 min. This work shows that easy-to-use common fluorescent dyes combined with photoluminescence spectroscopy methods can be used as an alternative method for the detection and quantification of nanoplastics in water environments and swarming magnetic nanorobots for efficient capture and removal. These methods hold great potential for future research to improve the quantification and removal of nanoplastics in water, and it will ultimately reduce their harmful impact on the environment and human health.

## 1 Introduction

Micro- and nanoplastics are an emerging type of organic persistent pollutant that is generated by the uncontrolled industrial human activity and the low environmental conscience of our society.^1−3^ Their presence in the environment is well-studied and reported, even more, they have been found in isolated places like alpine snow or the Arctic ice.^4,5^ Alarmingly, recent studies are finding these small plastic particles in a variety of living organisms.^6−9^ To date, micro- and nanoplastics have mainly been found in aquatic fauna,^6^ but also in birds;^7^ and more alarmingly, microplastics have been discovered in human placenta and blood.^8,9^ In the latter studies, microplastics have received most of the attention, but if microplastics are found it is unreasonable to believe that smaller nanoplastics are not present in the same environments.^10,11^ The challenge with nanoplastics is their reduced size, where currently applied methods for the observation of microplastics are not able to accurately detect nanoplastics.^11^ For this reason, it is of great importance to design a reliable and trustworthy method to detect and quantify the presence of nanoplastics in water.

Microplastics detection is a well-studied field with a vast variety and complexity of methods that have been developed for their detection. Some examples are, pyrolysis gas-chromatography/mass spectroscopy,^12,13^ Raman spectroscopy,^14−16^ and micro-Fourier-transform infrared spectroscopy.^17^ One of the most used methods in microplastics detection and quantification relies on the staining of plastics with the fluorescent hydrophobic dye Nile Red.^18,19^ This dye enables the observation and monitoring of a wide variety of plastic particles (polystyrene, polypropylene, and polyethylene, among others). On the other hand, applying different staining agents could monitor nanoplastics mixtures. Where 4-dimethylamino-4′-nitrostilbene would provide information on the nature of the polymer by varying the emissions wavelength according to the medium’s polarity, exhibiting a redshift as the polymer’s polarity increased.^20^

In such observation conditions, commonly, fluorescence microscopy of a filtered sample is used to detect microplastics, having a limitation in the resolution limit of such microscopes.^21^ For this reason, recent studies in the field^22,23^ are trying to avoid the use of microscopes in the detection process. Recently, the detection of 50 and 100 nm nanoplastics was achieved using a very specific biological media as dispersant.^22^ Other research articles present the possibility to use flow cytometry as an individual counting method for stained nanoplastics.^23^ In this case, there is a size limitation of the used particles, where smaller nanoplastics require modifications of the flow cytometry equipment initially designed for cells. Consequently, the development of a simple and reliable method to detect and quantify nanoplastics in pure water is important.

Not only is it important to detect nanoplastics but also it is of outermost importance to remove them from water environments. Nanorobots are used as a newly emerging type of pollutant removal; for dyes,^24,25^ oil–water separation,^26,27^ inorganic and organic pollutants,^28−30^ and microplastics capture and removal.^31−35^ Nanorobots are defined as nanosized structures that have automotive capabilities when applying a suitable source of energy. Typically, two main classes can be distinguished, chemically driven (fuel) and externally driven (electromagnetic field, light, or ultrasound).^34,36−38^ In the removal of plastic debris from the water, magnetic nanorobots are receiving growing attention,^39−41^ as their properties enable a simple separation procedure from an aqueous solution. Although there are reports of the capture and removal of microplastics, fewer studies are centering on nanoplastics.^42^

Here, we developed a new approach for the direct detection and quantification of nanoplastics and their remediation by applying magnetically navigated nanorobots. The detection and quantification are carried out via spectrofluorimetric techniques based on the specific staining of nanoplastics with Nile Red. Subsequently, the water cleanup is carried out using on-the-fly nanorobotics monitoring and removal of nanoplastics. For the demonstration of the applicability of the developed method for nanoplastic quantification, we employed magnetic nanorobots for the capture and removal of nanoplastics in water and evaluated their efficiency. The presented detection method in this work shows simple, easy-to-automate approaches for nanoplastic monitoring and removal, and it should replace currently used tedious and time-consuming methods. Distinguishing this work for the simplicity of the presented method and the ability to monitor tiny plastic particles in water environments. With a final aim to uncover the world of nanoplastic contamination, that is usually overseen or unable to be observed.

## 2 Materials and Methods

### 2.1 Materials

The sulfonated 200 nm polystyrene nanoparticles were purchased from Polyscience Inc. (Warrington, PA, USA). Iron (II, III) oxide nanopowder of spherical 50–100 nm nanoparticles (magnetite, 97% purity) and Nile Red for microscopy were purchased from Sigma-Aldrich (Merck, Germany). Ethanol 96% was purchased from Penta chemicals unlimited (Praha, Czech Republic).

### 2.2 Characterization

Nanorobots and nanoplastics were characterized using a TESCAN MIRA3 XMU SEM instrument equipped with an Oxford Instruments energy dispersive X-ray (EDS) detector. *Z*-potential in deionized water of both was found out using a Malvern Panalytical Zetasizer Ultra. The attachment of the nanoplastics to the nanorobots was studied with a Nikon Eclipse Ti2 inverted optical microscope coupled with a digital camera (Hamatsu C13440). The system was equipped with a TRITC filter (excitation at 532 nm, green light) and a CoolLED Pe-300 lite as the light source.

### 2.3 Locomotion Study

Nanorobots were navigated in aqueous solution with a transversal rotating magnetic field^43^ using a homemade setup.^44,45^ An alternating magnetic field of 5 mT with frequencies in intervals of 1 Hz from 0 to 5 Hz were used. The movement was recorded in videos of 20 fps for a duration of 20 s by using an inverted optical microscope (Nikon Eclipse Ts2R) equipped with a digital camera (Basler ace acA1920–155uc). The videos were processed using ImageJ software,^26^ from which the average velocity was obtained.

### 2.4 Detection of Nanoplastics

First, stock solutions of 95 mmol/L of Nile Red in ethanol and of 6 × 10^10^ beads/mL of nanoplastics in deionized water were prepared. All stock solutions were stored in a dark environment at 4 °C until use. The Nile Red stock solution (500 μL) was used to stain 5 mL of nanoplastics solution of different concentrations ranging from 0 to 280 × 10^8^ beads/mL. As prepared samples were left shaking for 20 h to enable staining the nanoplastics. Following this incubation period, the fluorescence spectra were obtained using Jasco spectrofluorometer FP-8300. For the observation the excitation wavelength was set to 560 nm, with an emission bandwidth of 5 nm. All measurements are performed at room temperature. The staining and observation of nanoplastics with Nile Red is meant to be a final step in the analysis procedure. It should be done under controlled laboratory conditions after extraction and isolation of the nanoplastics from any matrix.

### 2.5 Removal of Nanoplastics

For a typical nanoplastics removal experiment, nanoplastics colloidal solutions (110 × 10^8^ beads/mL) were treated with 0.8 mg/mL of nanorobots in 5 mL of water.^39^ The samples were placed in the magnetic field of a magnetic stirrer (500 rpm), and the capture of nanoplastics was monitored for 0, 20, 60, and 120 min. To determine the capture efficiency, the nanorobots were collected with a permanent neodymium magnet, and the supernatant was treated with Nile Red according to the staining procedure of nanoplastics detection. The removal experiments were carried out at room temperature.

## 3 Results and Discussion

### 3.1 Capture of Nanoplastics by Nanorobots

**Figure 1:**
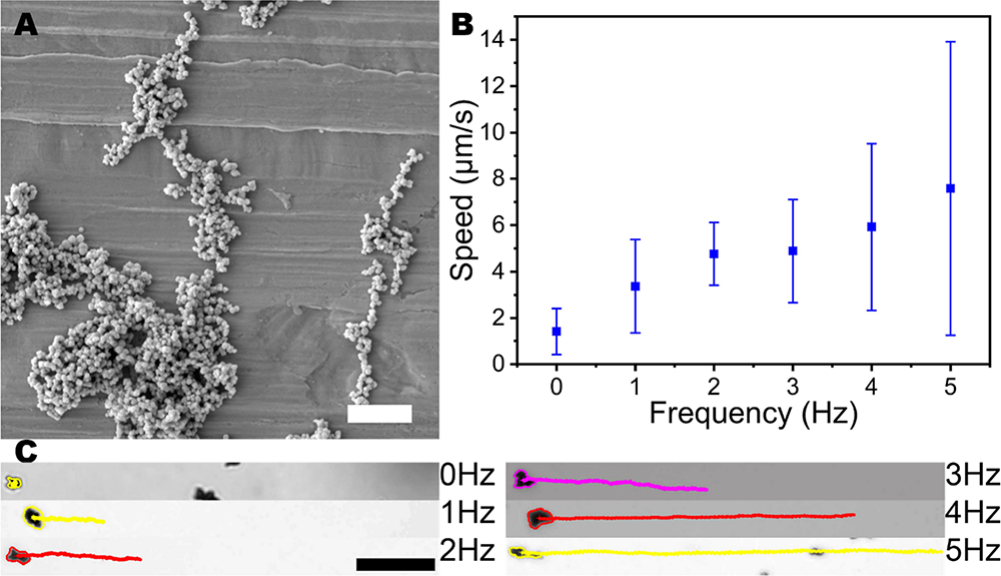
Characterization of the nanorobots. (A) SEM image of the employed nanorobots, scale bar 2 μm. (B) Average speed of nanorobots under 5 mT transversal rotating magnetic field at different frequencies. (C) Representative motion trajectories of the nanorobots collected for 20 s while applying a transversal rotating magnetic field of 5 mT, scale bar 20 μm.

The scanning electron microscopy (SEM) image in Figure 1A shows the morphology of the nanorobots. In this image, it can be observed that the nanorobots have a heterogeneous distribution of size and morphology (180 ± 90 nm, Supporting Information (SI) Figure S1). To characterize the nanorobots movement and control of motion, they were wirelessly actuated by a transversal rotating magnetic field.^44^ The action of such a field generates a tumbling motion on the robots; this rotation is translated into a unidirectional movement. The nanorobots average speed as a function of the frequency is represented in Figure 1B, a linear increase in the average speed can be observed in the studied range from 0 to 5 Hz. The typical trajectories of the nanorobots are presented in Figure 1C where it can be easily seen that higher frequencies generate larger displacement distances.

Subsequently, we utilized the nanorobots for nanoplastics capture. Well-defined sulfonated polystyrene nanoparticles with a diameter of 200 ± 10 nm (SI Figures S2 and S3) were used as a representative of nanoplastics. Surface functionalization with sulfo groups increases the stability of nanoplastics in water and provides them with a negative surface charge. DLS studies indeed showed that nanoplastics have a *Z*-potential of −67.5 ± 0.3 mV. As the nanorobots surface charge was of slightly positive *Z*-potential of 5.9 ± 0.9 mV (SI Figure S4), the electrostatic attraction between nanorobots and nanoplastics was possible.^46,47^ Previous research in this area has shown that due to the aging process that plastic particles undertake in the environment their *Z*-potential should be negative at neutral pH.^34,48^ Moreover, they have shown that for the removal of nanoplastics, a relevant interaction is the electrostatic one, which is highly dependent on the *Z*-potential of the involved particles. Based on this, we can assume that this kind of plastics with negative *Z*-potential, with the ability to interact electrostatically, will respond to the treatment in the same way. As a result, the pH of the extraction medium is of crucial importance.^49^ A way to limit the influence of the medium on the removal of nanoplastics while maintaining high rates of capture, the surface of nanorobots can be functionalized, for instance, the use of polydopamine has shown efficient microplastics capture.^32^

**Figure 2:**
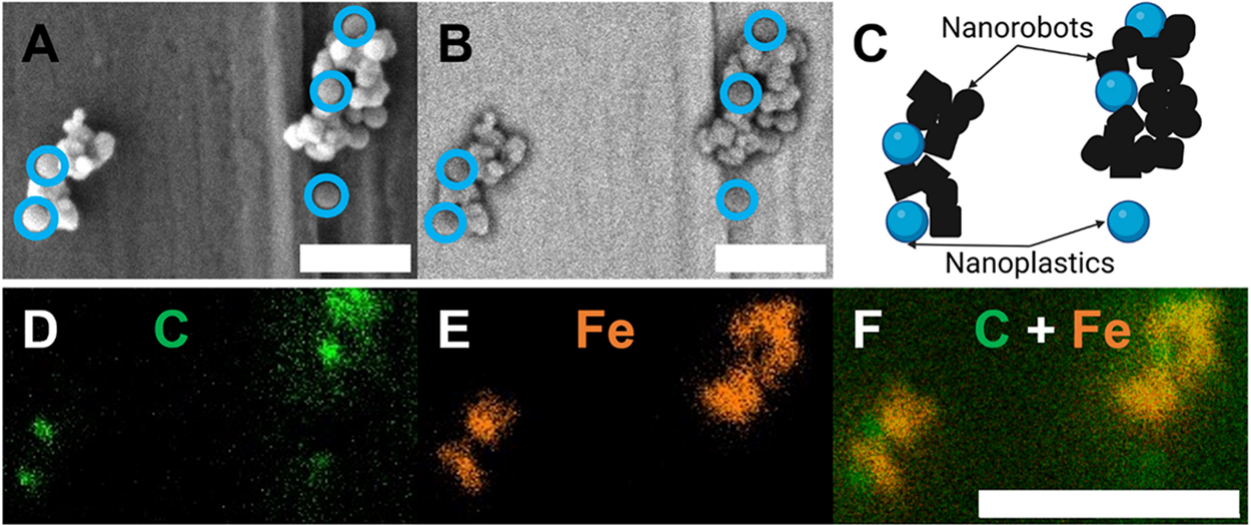
Proximity and interaction between nanorobots and nanoplastics. SEM images of a cluster: (A) secondary electrons and (B) backscattered electrons; circled particles in blue correspond to nanoplastics, scale bar of 1 μm. (C) Schematic representation of the cluster, in black the nanorobots and in blue the nanoplastics. EDS mapping shows the presence of carbon and iron (D) carbon map, (E) iron map, and (F) superposition of the previous two. Scale bar of 2 μm.

To demonstrate that the nanoplastics were attached to the nanorobots, SEM and EDS mapping were performed (Figure 2). Figure 2A shows the secondary electron (SE) image of the particle cluster; spherical ones can be associated with the nanoplastics and irregular ones with the nanorobots. Further confirmation can be found from the backscattered electron (BSE) image in Figure 2B. Given that the number of backscattered electrons generated by a sample increases with the atomic number of the components,^50^ the nanorobots appear brighter and the nanoplastics are darker in the BSE image. A schematic representation of this cluster can be seen in Figure 2C. The original micrographs of the captured nanoplastics without any labels are presented in Figure S5. To verify the nature of the observed particles, elemental mapping images were acquired by EDS. Figure 2D–F represents the mapping of the interest region and clearly shows that carbon atoms are concentrated in the regions where the nanoplastics are, confirming the previous electron microscopy images. The oxygen mapping can be observed in Figure S6 and more examples of the interaction between nanoplastics and nanorobots are shown in Figure S7. The SEM and EDS studies reveal the attraction and intimate contact between the studied particles, enabling the possibility of using nanorobots to capture and remove nanoplastics.

### 3.2 Spectrofluorimetric Method of Quantification of Nanoplastics

**Figure 3:**
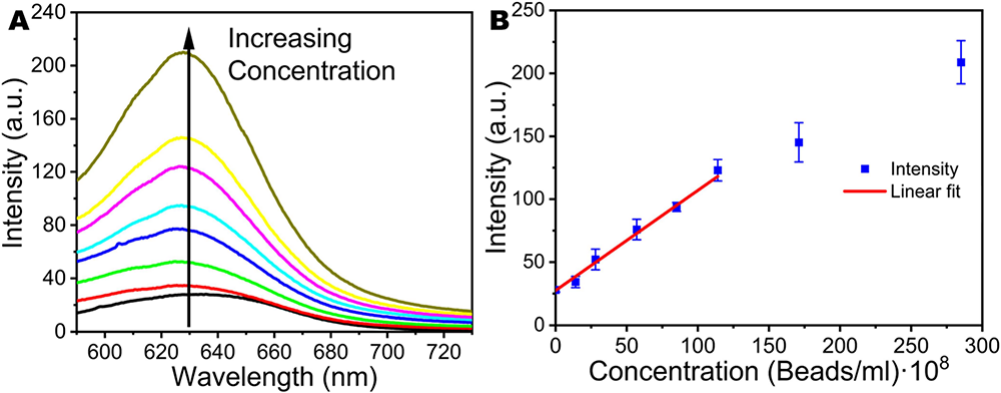
Photoluminescence study of labeled nanoplastics. (A) Recorded fluorescence spectra of the different plastic concentrations used for the calibration. Arrow represents growing concentration of nanoplastics. (B) Calibration curve of the spectrofluorometric concentration detection of labeled nanoplastics with Nile Red, with a linear trend for a given interval of concentrations.

Nile Red is a hydrophobic fluorescent dye that specifically binds to lipophilic materials and is strongly fluorescent in a lipophilic environment.^51^ This fluorophore has been used to detect micro- and nanoplastics, due to its specific hydrophobic attachment to plastics.^18,19^ Common epifluorescence microscopes and manual counting are used to determine the concentration of microplastics in a sample, having a drawback in the resolution limit, which does not allow the observation of nanoplastics. For this reason, we propose the use of an optical photoluminescence spectroscopy technique for the detection of nanoplastics. Here, the specific staining of nanoplastics by Nile Red is used to generate a fluorescence signal, which is measured with a spectrofluorometer. The recorded spectra are shown in Figure 3A, where it can be appreciated that higher concentrations of stained nanoplastics generate a higher signal. Figure 3B represents the relationship between the registered maximum (emission intensity at 630 nm) and the concentration of the nanoplastics.

From this data, it can be observed that there is a linear trend in the relationship between the intensity and the concentration range from 0 to 110 × 10^8^ beads/mL (*R*^2^ = 0.996). A solution with 0 beads/mL is taken as a control blank experiment of the developed method, and measurements with intensities in the vicinity of this spectrum are considered under the detection limit. Concentrations of nanoplastics higher than 110 × 10^8^ beads/mL present opaque solutions and enhanced scattering effects, setting the maximum concentration of the presented method. To monitor higher concentrations of nanoplastics in water, the most suitable methodology would be the dilution of the sample in the range of the presented calibration range. Further research should focus on the analysis of different sizes of nanoplastics and mixtures with a broad size distribution of plastic particles. The presented method of detection is not intended to monitor microplastics in the water solution; the higher surface of microplastics will interfere with the measurements centering on nanoplastics. A possible investigation direction would be an initial separation by size, obtaining a narrow distribution of nanoplastics. To conclude, the results show the possibility to quantify concentrations of nanoplastics in water in the range of 10^8^–10^10^ beads/mL.

### 3.3 Removal of Nanoplastics with Nanorobots

**Figure 4:**
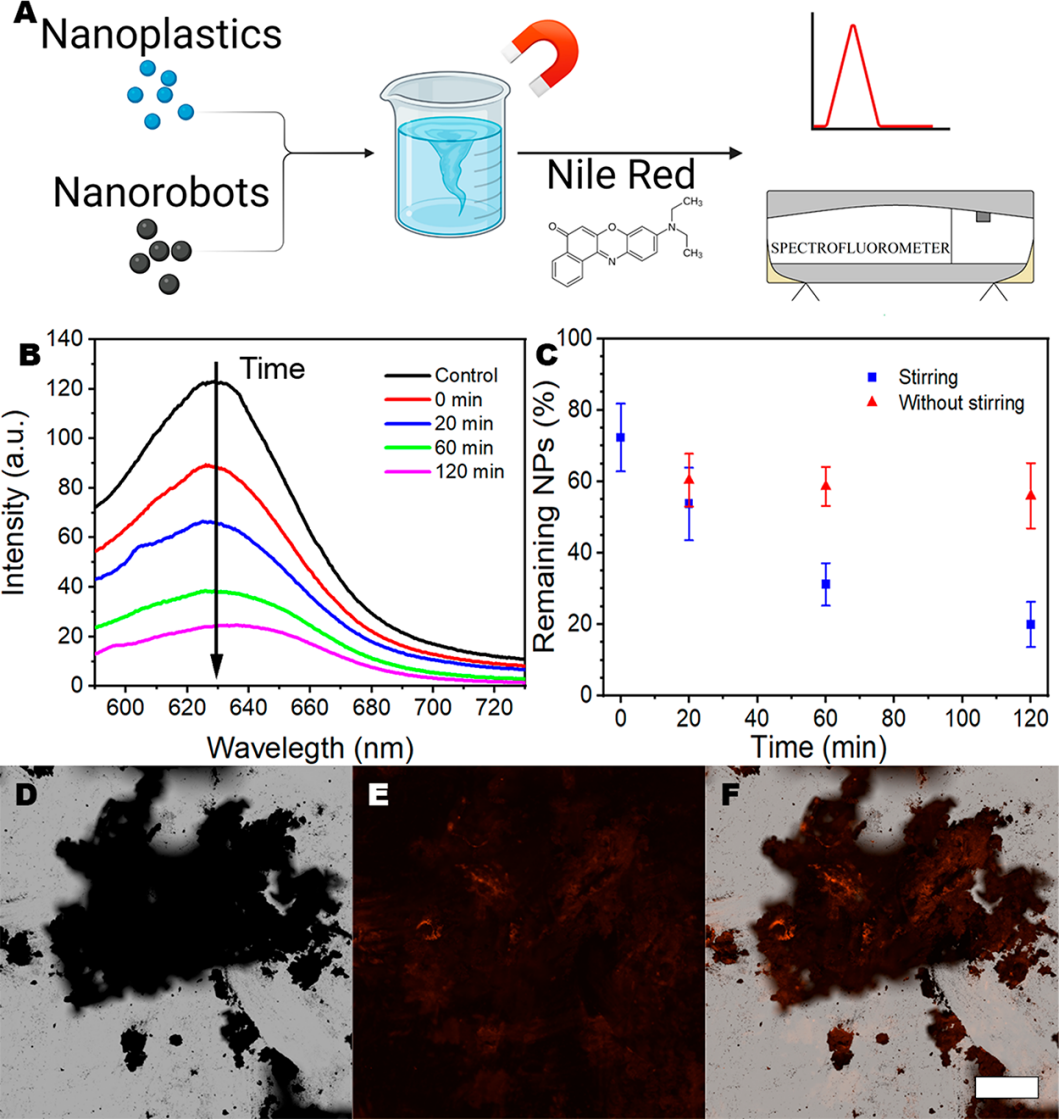
(A) Schematical representation of the performed work, with the nanorobotic removal and the use of Nile Red for observation. (B) Intensity of a solution of stained nanoplastics after different times of removal treatment with magnetite. (C) Remaining % of nanoplastics from the different removal treatments. Study of the removal of nanoplastics with magnetite from water. Fluorescence microscopy images of magnetite with stained PS: (D) bright field, (E) green excitation, and (F) combined; scale bar 200 μm.

For the removal and posterior efficiency computation, nanorobots and nanoplastics were mixed by using the rotating magnetic field in different time intervals ranging from 0 to 120 min. This ensured that the nanoplastics were trapped with nanorobots. Subsequently, the trapped nanoplastics by the nanorobots were removed from the solution by an external magnetic field in the form of a permanent neodymium magnet. The remaining solution (supernatant) was collected and treated with Nile Red to determine the concentration of unremoved nanoplastics. (Figure 4A). The removal efficiency was evaluated by calculating the concentration of the remaining nanoplastics in the supernatant by applying the calibration curve shown in Figure 3B. Figure 4B shows the spectra of the removal experiments. Clearly, longer capture time leads to improved efficiency of nanoplastics removal, as demonstrated by the decreasing intensity signal. As Figure 4C shows, by only mixing the nanorobots with the nanoplastics and separating them with an external magnet, 30% of the contaminant was removed. It is worth noting that after treating the nanoplastics with nanorobots for 120 min, the intensity signal of the remaining nanoplastics is in the vicinity of the detection limit. As a result, it is indicated that the nanorobots can remove more than 90% of the present nanoplastic contamination within 120 min. In previous studies, close values were obtained for the removal of microplastics with similar systems.^39,47,52^ Given the similarities in these conditions and previously reported ones, we expect that the removal and monitoring of other polymers (polyethylene terephthalate, polypropylene, polyethylene, among others) will be possible.^53^

Besides the similarities with previously presented methods, in this work it is possible to determine the removal rate for nanoplastics. In addition, it is important to notice that the movement of the nanorobots is of utmost importance in the removal of nanoplastics. The control experiment of nonmoving (stationary) nanorobots in the presence of nanoplastics resulted in negligible removal efficiency, as expected. This is clear proof that the motion of the nanorobots results in much higher mass transfer and subsequent active trapping of nanoplastics, a key component for the efficient trading and removal of nanoplastics via nanorobotic tools. (Figure 4C) Overall, we propose a simple and fast method to detect nanoplastics, in comparison to currently used micro-FTIR, micro-RAMAN, and mass spectroscopy methods.^12−15,17^ Using the proposed method demonstrates that nanorobots can remove nanoplastics from water in short treatment times. Based on previous research on this topic,^28,54^ nanoplastics can be detached from the nanorobots in ethanol. These findings allow for the potential recycling of the nanorobots and their reuse in further experiments.

To further corroborate that the nanoplastics are being trapped by the nanorobots and not by the surface of the container, the presented nanoplastic staining approach was used. To do so, a solution of stained nanoplastics in water was mixed with nanorobots for 120 min. Afterward, the magnetic nanorobots were separated by using a permanent neodymium magnet and left to dry at ambient temperature. Following, a fluorescent microscope was used to monitor the attached stained nanoplastics to the nanorobots’ surface. Figure 4D–F shows the capture of nanoplastics by the nanorobots. From the combined image, it can be appreciated that not only the nanoplastics are getting stained but also that the nanoplastics are attached to the nanorobots; further confirming the previous findings from SEM and EDS. To prove that Nile Red was not staining the nanorobots, images of combined bright-field and green excitation were analyzed (SI Figure S8A). In addition, to check that the nanoplastics do not present autofluorescence, they were mixed for 120 min with the nanorobots and observed with the fluorescent microscope (SI Figure S8B). Both control cases show no fluorescence signal while keeping the camera’s sensitivity and exposition time as in the experiments summarized in Figure 4D–F. Corroborating that in Figure 4F, the fluorescence is coming from the stained nanoplastics with Nile Red attached to the nanorobots’ surface.

The presented method is straightforward to implement in a chemical laboratory for the detection of nanoplastics, with the expectation that with further development it will overcome the challenges that current methodologies are faced with. To compare with similar studies, Yu et al.^14^ detected 30 nm polymer particles, using a very complex setup with a complicated surface to obtain the desired surface-enhanced Raman spectroscopy effect. On the other hand, in a study by Bianco et al.^23^ the detection limit was 600 nm, but a complex data treatment and expensive equipment were required to obtain the best results. Future work in our methodology will aim at the improvement of the staining process for the better observation of even smaller concentrations of nanoplastics in water, where it is believed that the design of new and improved fluorescent dyes is a suitable investigation direction. We expect that future developed and tested staining agents will be able to specifically stain different types of polymers, broadening the application perspectives of the method and allowing for the observation of a mixture of polymers in water. It is expected that with further optimization and development, this method will be easily implemented in laboratory analysis to monitor the presence of nanoplastics, a contaminant that is challenging to detect with conventional methodologies.

## 4 Conclusions

We developed a spectroscopic method for the quantification of nanoplastics and a nanorobotic tool to capture and remove nanoplastics. The presented method has proven to be a suitable tool for the observation of nanoplastics in a water solution. Specific staining of nanoplastics with Nile Red and its subsequent spectrofluorimetric detection have proven to be a versatile method to monitor this environmental pollutant. To evaluate the applicability and efficiency of the presented detection method, magnetic nanorobots were used to capture and remove the nanoplastics from aqueous environments. As a result, it is reported that the nanorobots were able to capture and remove suspended nanoplastics from water within 120 min with 90% efficiency. In summary, fluorescent staining of nanoplastics and photoluminescence techniques for observation have proven to be a method that is useful, simple, easy to scale up, and fast to detect and quantify the presence of nanoplastics in water. It is expected that this method will find wide use in the field of nanoplastic detection and removal.

## Supporting Information Available

Size distribution of nanorobots and nanoplastics, SEM image of nanoplastics, *Z*-potential of nanoplastics and nanorobots, original micrograph of captured nanoplastics, EDX analysis, and multichannel fluorescence images (PDF)
