# Supplementary material for: On-the-Fly Monitoring of the Capture and Removal of Nanoplastics with Nanorobots
Source: ACS Nanosci Au. 2024 Apr 9;4(4):243–9. doi: 10.1021/acsnanoscienceau.4c00002 (PMC11342339; doi:10.1021/acsnanoscienceau.4c00002)
Supplement: Supplementary file 1 — ng4c00002_si_001.pdf [file ng4c00002_si_001.pdf]

SUPPORTING INFORMATION

On-the-fly monitoring of the capture and removal of nanoplastics with nanorobots

Dean I. Velikov[1], Anna Jancik-Prochazkova[1] & Martin Pumera[1,2,3*]

1 Future Energy and Innovation Laboratory, Central European Institute of Technology, Brno University of Technology, Purkyňova 123, 612 00 Brno, Czech Republic.

2 Advanced Nanorobots & Multiscale Robotics Laboratory, Faculty of Electrical Engineering and Com-puter Science, VSB - Technical University of Ostrava, 17. listopadu 2172/15, 708 00 Ostrava, Czech Re-public

3 Department of Medical Research, China Medical University Hospital, China Medical University, No. 91 Hsueh-Shih Road, 406040 Taichung, Taiwan.

email: pumera.research@gmail.com

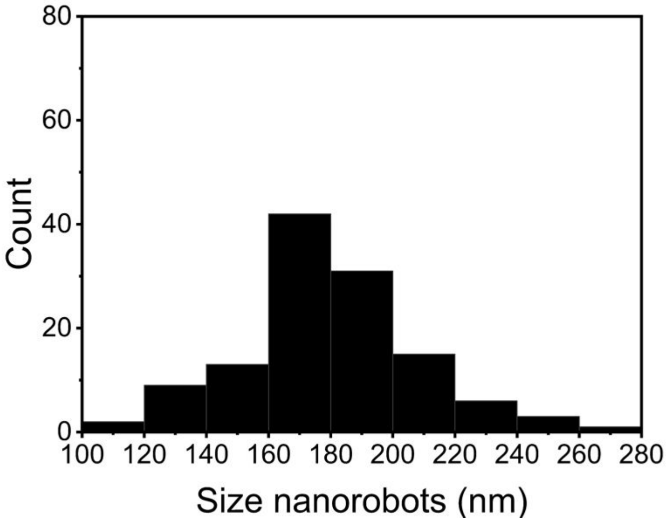

Figure S1: *Size distribution of the nanorobots obtained from the SEM images.*

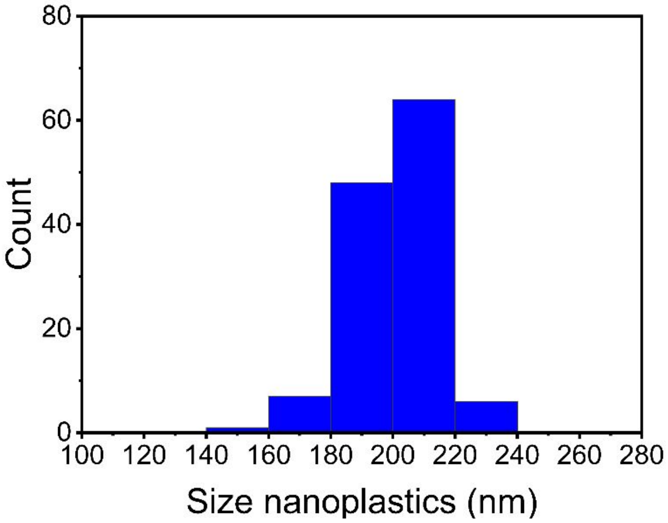

Figure S2: *Size distribution of the nanoplastics obtained from the SEM images.*

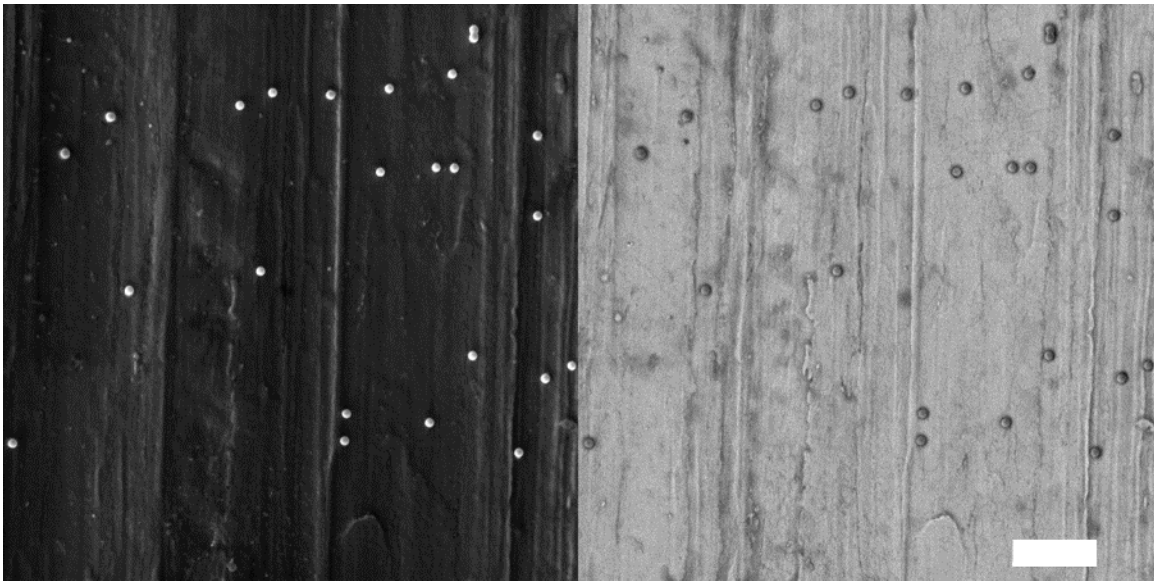

Figure S3: *SEM characterization of 200 nm polystyrene nanoparticles, nanoplastics. SE image on the left and BSE image on the right. Scale bar 1 µm.*

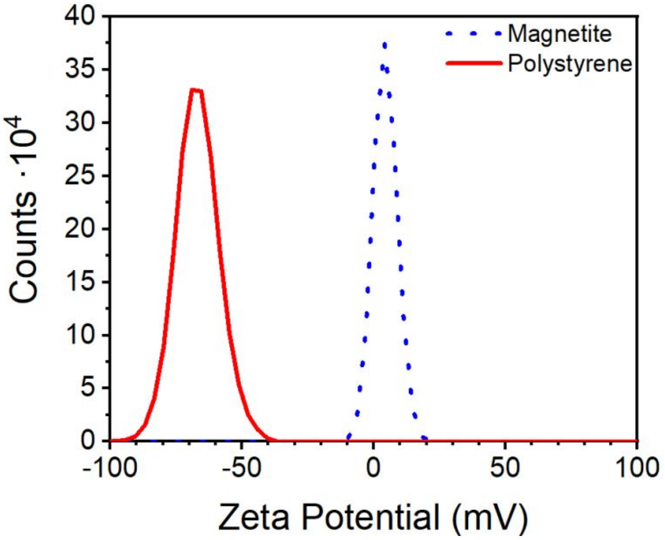

Figure S4: *DLS study of nanoplastics (polystyrene) and nanorobots (magnetite). Graph showing the Z-potential of the corresponding particles.*

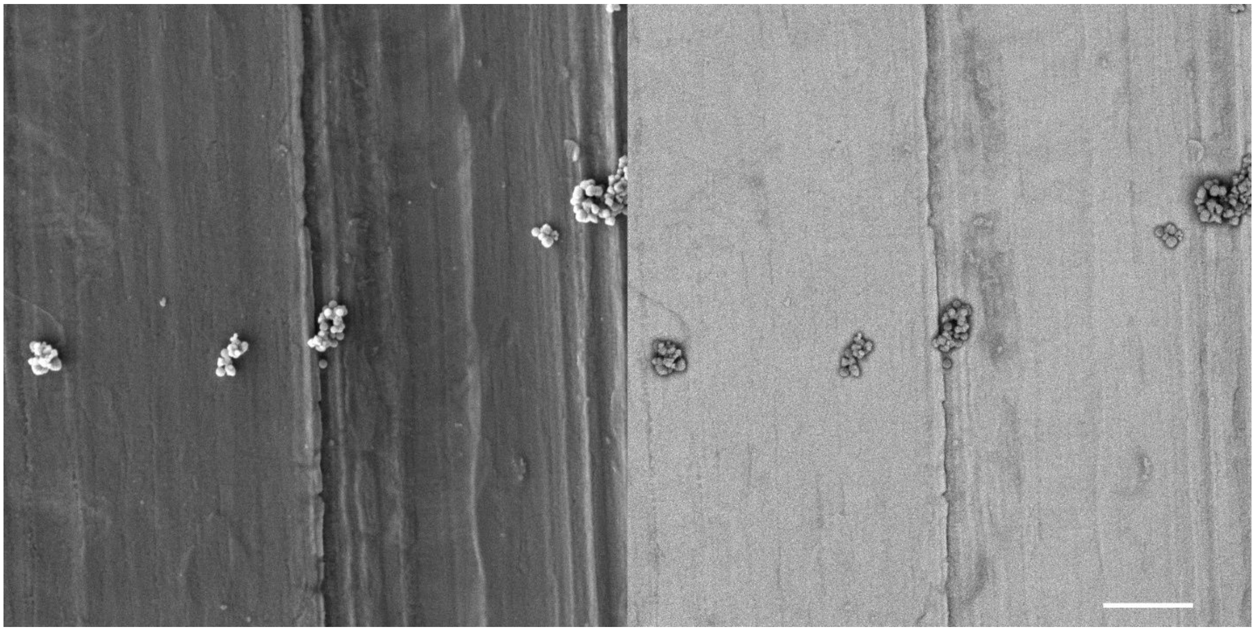

Figure S5: *Original micrographs of the captured nanoplastics without any labels from the presented cluster in Figure 2 in the manuscript. Scale bar 2 µm.*

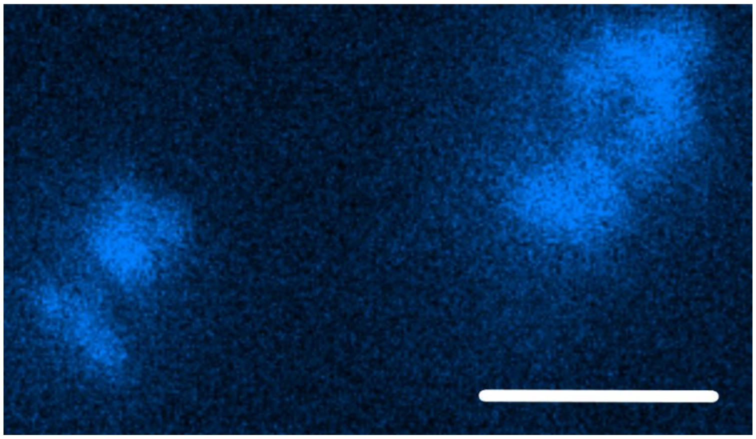

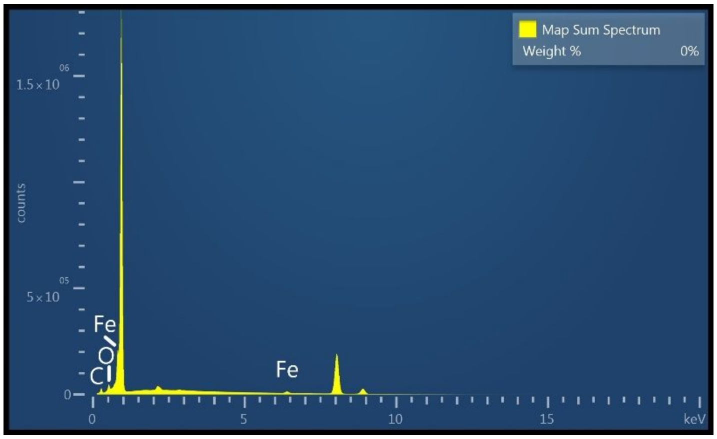

Figure S6: *Oxygen mapping of the nanorobots/nanoplastics cluster presented in Figure 2 of the manuscript. Scale bar 2 µm (left). EDS spectra of the detected elements (Fe, C and O (right).*

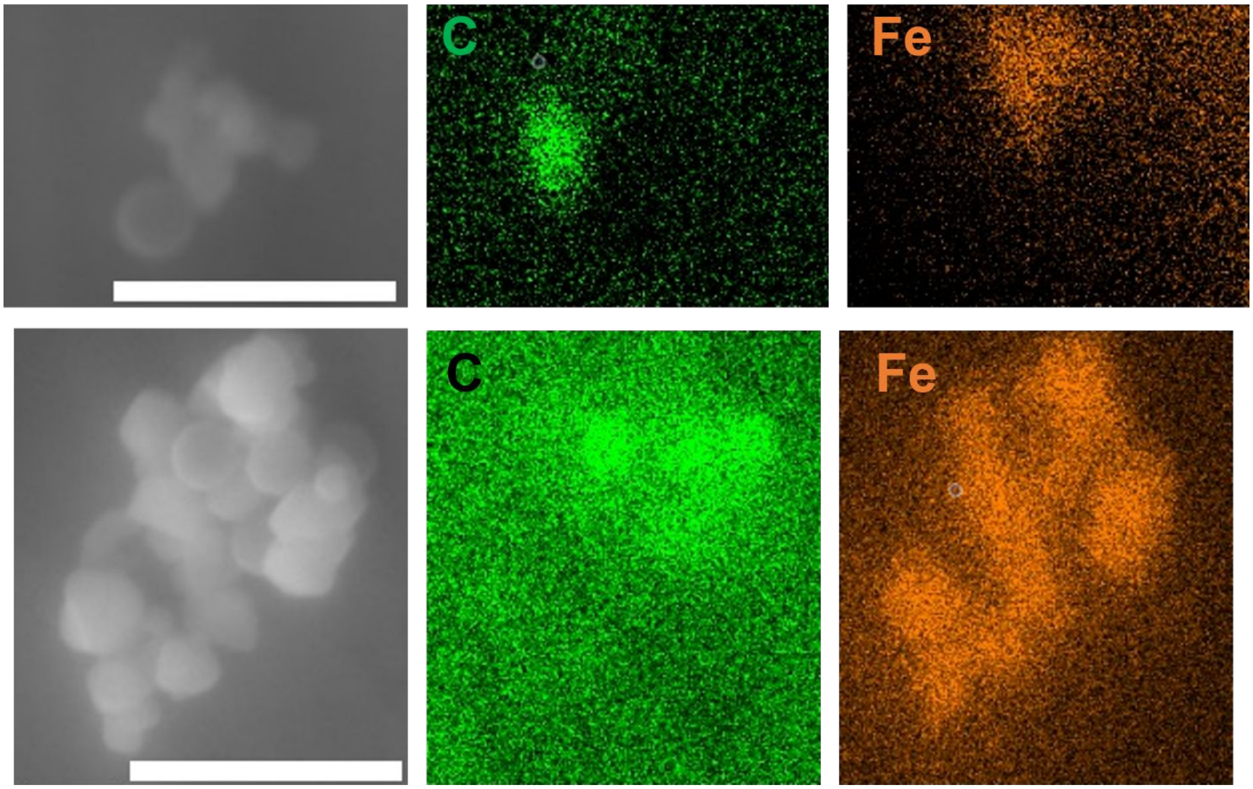

Figure S7: *More examples of SEM and EDS mappings of Carbon and Iron of different clusters of nanoplastics and nanorobots. Scale bar 2 µm*

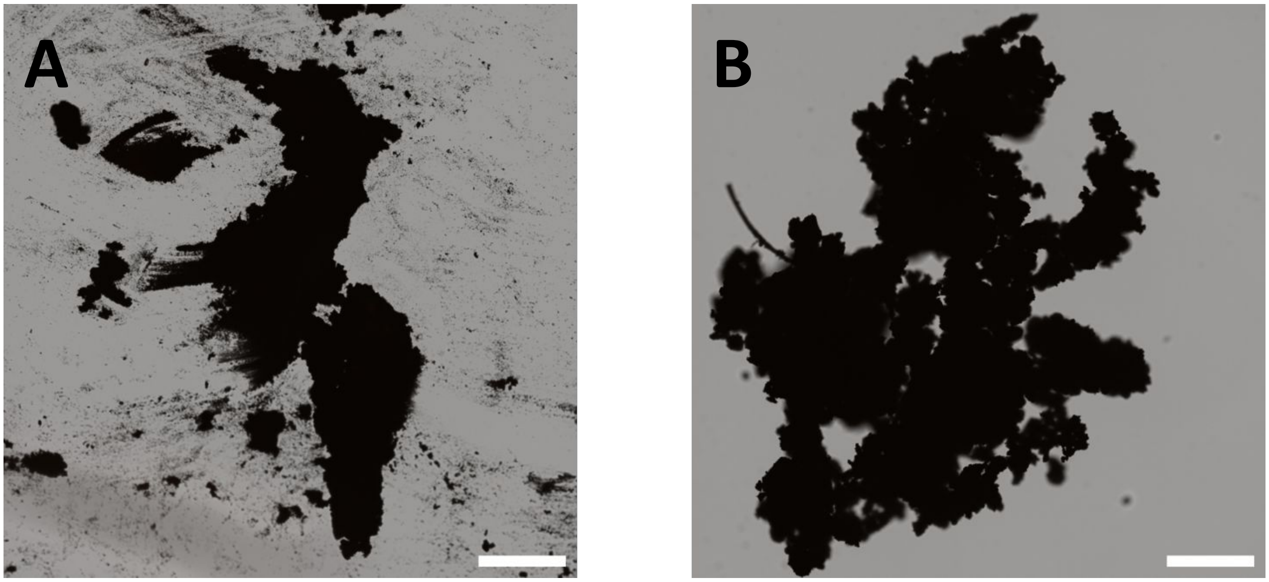

Figure S8: *Multichannel bright field and green excitation. Magnetite after 2h in a water solution of Nile Red(left). Magnetite with unstained polystyrene nanoplastics (right). Scale bar 200 µm.*
